# Errata

**Published:** 1884-10

**Authors:** 


Errata.—In Dr. Wells' articles the following errata occur: Page 109, 20th line, for *part* read *fact*; page 235, 22d line, for *part* read *fact*.

